# Ethanol production from mixtures of wheat straw and wheat meal

**DOI:** 10.1186/1754-6834-3-16

**Published:** 2010-07-02

**Authors:** Borbála Erdei, Zsolt Barta, Bálint Sipos, Kati Réczey, Mats Galbe, Guido Zacchi

**Affiliations:** 1 Lund University, Department of Chemical Engineering, P.O. Box 124, SE-221 00 Lund, Sweden; 2 Budapest University of Technology and Economics, Department of Applied Biotechnology and Food Science, 1111 Budapest, Szt. Gellért tér 4, Hungary

## Abstract

**Background:**

Bioethanol can be produced from sugar-rich, starch-rich (first generation; 1G) or lignocellulosic (second generation; 2G) raw materials. Integration of 2G ethanol with 1G could facilitate the introduction of the 2G technology. The capital cost per ton of fuel produced would be diminished and better utilization of the biomass can be achieved. It would, furthermore, decrease the energy demand of 2G ethanol production and also provide both 1G and 2G plants with heat and electricity. In the current study, steam-pretreated wheat straw (SPWS) was mixed with presaccharified wheat meal (PWM) and converted to ethanol in simultaneous saccharification and fermentation (SSF).

**Results:**

Both the ethanol concentration and the ethanol yield increased with increasing amounts of PWM in mixtures with SPWS. The maximum ethanol yield (99% of the theoretical yield, based on the available C6 sugars) was obtained with a mixture of SPWS containing 2.5% water-insoluble solids (WIS) and PWM containing 2.5% WIS, resulting in an ethanol concentration of 56.5 g/L. This yield was higher than those obtained with SSF of either SPWS (68%) or PWM alone (91%).

**Conclusions:**

Mixing wheat straw with wheat meal would be beneficial for both 1G and 2G ethanol production. However, increasing the proportion of WIS as wheat straw and the possibility of consuming the xylose fraction with a pentose-fermenting yeast should be further investigated.

## Background

The use of bioethanol can reduce our dependence on fossil fuels, while at the same time decreasing net emissions of carbon dioxide, the main greenhouse gas [1,2]. However, large-scale production of bioethanol is being increasingly criticized for its use of food sources as raw material. Brazil's bioethanol production consumes large quantities of sugar cane, while in the USA, corn is used [3]. Other starch-rich grains, such as wheat and barley, are mostly used in Europe [4]. The use of such sugar-rich feedstock causes the escalation of food prices, owing to competition on the market [5,6]. Therefore, future expansion of biofuel production must be increasingly based on bioethanol from lignocellulosic materials, such as agricultural byproducts, forest residues, industrial waste streams or energy crops [7,8]. These feedstocks, which are being used in second-generation (2G) bioethanol production, are abundant, and their cost is lower than that of food crops [9]. In Europe, wheat straw has the greatest potential of all agricultural residues because of its wide availability and low cost [10].

To efficiently utilize lignocellulosic products, pretreatment is required to hydrolyse the hemicelluloses to make the celluloses more accessible to the enzymes. One of the most suitable kinds of pretreatment for lignocellulosic material is steam explosion [11]. Combining steam explosion with acid catalysts is considered one of the most promising techniques for the commercialization of the process [12]. Several studies have shown that impregnation of wheat straw with small amounts of H_2_SO_4 _before steam pretreatment results in improved sugar yields [13,14].

During pretreatment, several sugar degradation products such as 5-hydroxymethyl-furfural (HMF) and furfural (degradation products of hexoses and pentoses, respectively), weak organic acids and phenolic compounds from lignin degradation are released into the hydrolysate, and have been shown to inhibit both yeast [15,16] and enzymes [17]; however, these compounds affect cell growth more than ethanol formation. It has also been shown by Larsson *et al*. that the ethanol yield in the presence of several inhibitors decreased only slightly compared with the reference fermentation [18]. Furthermore, the addition of weak acids has an intense inhibitory effect on growth of *Saccharomyces cerevisiae*, but leads to increased ethanol yield at low concentrations [19,20]. Therefore, we hypothesized that mixing starch hydrolysate with the lignocellulosic stream would dilute the inhibitor concentration in the cellulose hydrolysate and probably improve the fermentation, and at the same time, the presence of inhibitors might also improve the ethanol yield from the starch fraction.

To obtain efficient ethanol fermentation with *S. cerevisiae*, numerous nutrients, including trace metals and vitamins, are required during the process. Chemicals contribute significantly to the cost of large-scale production [21]; although it was not in the scope of this study to investigate this, their use should thus be minimized. Wheat hydrolysate, which is relatively cheap compared with chemicals, has been proven to be a potential supplement for lignocellulosic hydrolysate, because it is not only a sugar-containing material, but is also a complex nutrient source [22,23].

The production cost of ethanol is not only dependent on the yield but also on the concentration of ethanol in the fermentation broth, because of the high energy demand in the distillation step. In this step, the ethanol concentration in the broth after fermentation is increased to 94% using two stripper columns and a rectification column, which are heat-integrated by operating at different pressures. A significant increase in energy demand is observed at an ethanol concentration below 4% [24]. A higher ethanol concentration can be achieved in the broth by adding starch-rich material to the lignocellulosic process, leading to a lower energy demand in distillation, thus reducing the production cost.

The aim of this study was to evaluate the simultaneous saccharification and fermentation (SSF) of mixtures of cellulosic material (steam pretreated wheat straw; SPWS and presaccharified wheat meal (PWM). The effect on ethanol concentration and ethanol yield of varying the proportions of starch and cellulose fraction in SSF was investigated and compared with the pure starch and pure cellulose alternatives.

## Methods

**Figure 1:**
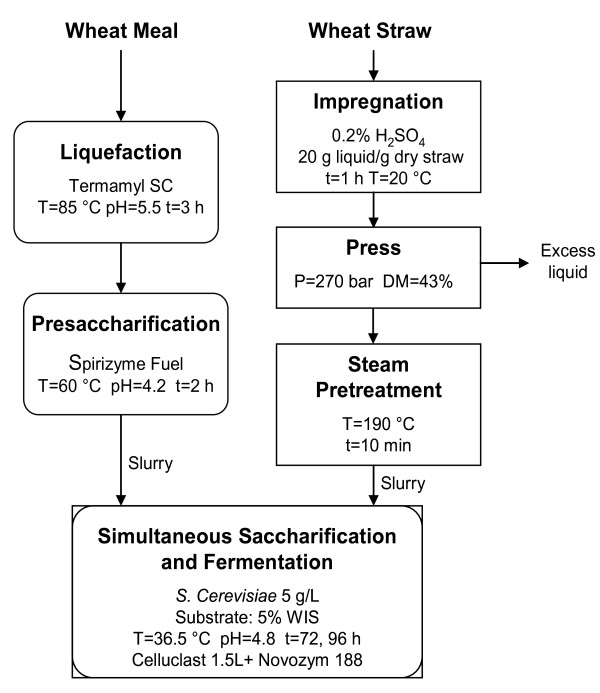
**Experimental procedure used to assess the effects of mixtures of wheat straw and wheat meal on simultaneous saccharification and fermentation (SSF)**.

The experimental procedure is illustrated in Figure 1. The wheat meal was subjected to liquefaction and supplemented with α-amylase, followed by 2 hours of presaccharification with amyloglucosidase. The entire wort (PWM) was used in SSF. The wheat straw was impregnated with a weak sulfuric acid solution and then squeezed using a hydraulic press. The pressed material was pretreated in a steam pretreatment unit and the whole slurry was then mixed with the PWM. The two materials were mixed in different proportions to investigate the effects on the ethanol yield and the ethanol concentration.

### Raw materials

Wheat straw was kindly provided by Lunds Civila Ryttarförening (Lund, Sweden). It was chopped in a hammer mill, sieved to obtain pieces of 2-10 mm, and then stored at room temperature before pretreatment. Wheat meal (dry-milled grain) with an average particle size of 2.5 mm was kindly provided by Sileco (Halland, Sweden) and stored at 5°C before use.

### Enzymes

α-Amylase (Termamyl^® ^SC; Novozymes A/S, Bagsværd, Denmark) and amyloglucosidase (Spirizyme^® ^Fuel; Novozymes) amylolytic enzymes were used for starch liquefaction and saccharification, respectively. The amylolytic activity of these enzymes were not measured, because they were loaded based on their weight, as it is recommended by the manufacturer [25]. In the SSF experiments, cellulase (Celluclast 1.5 L) and β-glucosidase (Novozym 188) enzyme preparations (both Novozymes) were used. Celluclast 1.5 L had an activity of 65 filter paper units (FPU)/g, measured using the IUPAC protocol [26], and 33 IU/g β-glucosidase activity according to the method of Berghem and Petterson [27]. Novozym 188 had a β-glucosidase activity of 350 IU/g.

### Compositional analysis

The carbohydrate and lignin contents of the raw wheat straw, the starch-free fibre, and the solid fraction of the pretreated wheat straw were determined according to the standard National Renewable Energy Laboratory (NREL) method [7,28]. Finely ground samples were treated with 72% H_2_SO_4 _for 1 h at 30°C, then diluted to 4% H_2_SO_4 _and autoclaved for 1 hour at 121°C. Sugar contents were analysed with high performance liquid chromatography (HPLC) (LC-10AD; Shimadzu, Kyoto, Japan), acid-insoluble lignin was measured by weighing after overnight drying at 105°C, and acid-soluble lignin was determined by spectrophotometry using a wavelength of 240 nm. Each sample was analysed in duplicate.

The liquid fraction of the SPWS and the supernatant after fermentation were analysed for total sugar content according to an NREL procedure [29]. In this method, the sample is treated with 4% H_2_SO_4 _at 121°C for 1 h, and then analysed by HPLC.

The fraction of acid-insoluble ash was determined after the two-step acid hydrolysis described above, and again on the ash of the residue. Both samples were heated at 550°C until the sample weight remained constant. Total ash refers to the inorganic part of raw material or solid fraction after pretreatment.

To determine the starch content, the wheat meal was subjected to a two-step enzymatic hydrolysis consisting of liquefaction and saccharification. All batches were hydrolysed using a 7 L evaporator (Rotavapor^® ^R-153; Büchi Labortechnik AG, Flawil, Switzerland). The dry matter content was set to 35%. In the first step, wheat meal slurry supplemented with 0.5 g/kg dry matter (DM) and Termamyl^® ^SC was liquefied at 85°C, pH 5.5, for 3 h. In the second step, Spirizyme^® ^Fuel was added at a ratio of 0.5 mL/kg DM at pH 4.2, and the slurry was treated at 60°C for 24 h to ensure total starch hydrolysis. The wort was filtered and the glucose content of the supernatant was measured using HPLC. The washed solid residue is referred to as the starch-free residue (SFR).

### PWM

Wheat meal was presaccharified as described above, except that the duration of saccharification was 2 h instead of 24 h. PWM was then used in SSF.

### SPWS

The wheat straw was immersed in an aqueous solution of 0.2% H_2_SO_4 _at a liquid:dry straw weight ratio of 20. It was stored in sealed buckets for 1 h, and was then squeezed in a manual 3 L press (Fisher Maschinenfabrik Gmbh, Burgkunstadt, Germany) to an average dry matter content of 43%. Steam pretreatment was performed in a unit (described previously [30]) comprising a 10 L pressurized vessel, with a flash cyclone in which the pretreated material was released and collected. Previously optimized conditions for wheat straw [14] were used; that is, the temperature was maintained at 190°C for 10 min using saturated steam. Each batch that was fed into the reactor was 600 g wet weight. The steam-pretreated wheat straw (SPWS) was then subjected to SSF.

### SSF

SSF experiments were performed in 2 L laboratory fermentors (Infors AG, Bottmingen, Switzerland) with a final working weight of 1.4 kg. PWM, SPWS slurry and various mixtures of these (denoted as mixtures A, B, C and D) were used as substrates in SSF with a total water-insoluble solids (WIS) content of 5%. The PWM:SPWS WIS ratios for the mixtures investigated were 0.8:4.2, 1.5:3.5, 2.0:3.0 and 2.5:2.5, respectively. When SSF was performed on pure PWM, the WIS content was set to 2.8% to restrict the ethanol concentration to 60 g/L. Details of the substrates can be found in Table 1. SSF experiments were performed using Celluclast 1.5 L and Novozym 188, at dosages of 15 FPU/g glucan and 17 β-glucosidase IU/g glucan, respectively. SSF of pure PWM was not supplemented with Celluclast 1.5 L or Novozym 188. *S. cerevisiae *(ordinary baker's yeast; Jästbolaget AB, Stockholm, Sweden) was suspended in sterilized water and added to the fermentor at a concentration of 5 g DM/L. As nutrients, (NH_4_)_2_HPO_4_, MgSO_4_·7 H_2_O and yeast extract were used at concentrations of 0.5, 0.025 and 1.0 g/L, respectively. The fermentor was loaded with the SPWS and the nutrients, which were sterilized separately at 121°C for 20 minutes, but the PWM was not sterilized because the starch and the enzymes already added would have been damaged at these conditions. SSF was performed at 36.5°C, and the pH was maintained at 5 ± 0.2 by addition of 10% NaOH solution. The experiments were run for 72 h in the case of pure PWM, and for 96 h in the case of SPWS or mixtures of the substrates. All samples withdrawn were filtered through 0.2 μm filters before being analysed by HPLC.

**Table 1:** Details of the substrates used in the SSF experiments

Substrate	WIS			Glucose equivalents			
	
	Total	PWM	SPWS	PWM		SPWS	
				
				WIS	Liquid	WIS	Liquid
SPWS	5	--	5.0	--	--	3.7	0.2
Mixture A	5	0.8	4.2	0.1	2.5	3.2	0.2
Mixture B	5	1.5	3.5	0.3	5.0	2.6	0.1
Mixture C	5	2.0	3.0	0.4	6.6	2.2	0.1
Mixture D	5	2.5	2.5	0.5	8.3	1.9	0.1
PWM	2.8	2.8	--	0.5	9.4	--	--

PWM = presaccharified wheat meal; SPWS = team-pretreated wheat straw; WIS = water-insoluble solids.

Data are presented as percentage of working weight.

### Analysis of sugars, ethanol and byproducts

The content of reducing sugars was measured colorimetrically using dinitrosalicylic acid, according to Miller's method [31]. The liquid fractions from pretreatment, samples from acid hydrolysis and the supernatants of SSF broth were analysed by HPLC, in a chromatograph equipped with a refractive index detector. Cellobiose, glucose, mannose, xylose, galactose and arabinose were separated on an ion-exchange column (Aminex HPX-87P; Bio-Rad Laboratories, Hercules, CA, USA) at 85°C. Ultrapure water was used as eluent at a flow rate of 0.6 mL/min. Lactic acid, glycerol, acetic acid, ethanol, HMF and furfural were separated (Aminex HPX-87H column; Bio-Rad Laboratories) at 65°C. The eluent was 0.005 M H_2_SO_4 _at a flow rate of 0.5 mL/min.

## Results and Discussion

The ethanol yields were calculated as a percentage of the maximal theoretical yield for glucose (0.51 g/g) that could have been produced if all the glucose present in the slurry and the PWM, including both monomers and oligomers in the liquid and glucan fibres in the WIS, had been converted to ethanol. The theoretical amount of glucose released during the hydrolysis was calculated by multiplying the amount of glucan by 1.11.

### Material composition and pretreatment

The composition of the raw materials is shown in Table 2. The DM content of wheat meal consists of 72.7% starch and 24.3% SFR, showing that this part of the crop could also be an important source of lignocellulose. Several studies have investigated ways in which the ethanol yield could be enhanced by utilizing this part of the crop [32,33]. However, pretreatment is required to facilitate the enzymatic hydrolysis of SFR fibres, which was not performed in the present study. The compounds determined in SFR constituted about 63% of the DM.

**Table 2:** Composition of raw wheat straw and wheat meal, in % of DM, including breakdown of the starch-free residue.

Component	Percentage of DM, mean ± SD	
	
	Wheat meal	Raw wheat straw
Starch	72.7	--
SFR^a^	24.3	--
	% of SFR^a^	
Glucan	17.5 ± 0.1	38.8 ± 0.5
Mannan	BDL	1.7 ± 0.2
Xylan	14.4 ± 0.0	22.2 ± 0.3
Galactan	1.6 ± 0.0	2.7 ± 0.1
Arabinan	8.5 ± 0.0	4.7 ± 0.1
ASL^b^	3.1 ± 0.0	2.4 ± 0.0
AIL^c^	15.1 ± 3.0	16.1 ± 0.1
Total ash	2.3 ± 0.3	5.8 ± 0.1
AIA^d^	BDL	2.4 ± 0.4

Where standard deviations are presented, these were calculated from duplicate measurements on the same sample.

^a^Starch-free residue.

^b^Acid-soluble lignin.

^c^Acid-insoluble lignin.

^d^Acid-insoluble ash.

BDL = below detection limit; DM = dry matter.

**Figure 2:**
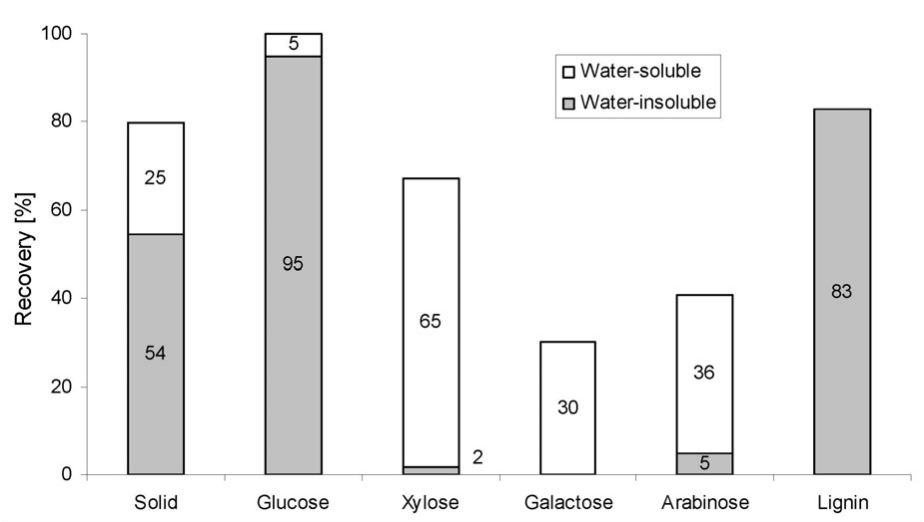
**Recovery of solids, sugars and water-insoluble lignin**. Percent of the theoretical, after steam pretreatment of wheat straw at 190°C for 10 minutes with 0.2% H_2_SO_4_.

The total solids recovery by the steam pretreatment was 79%. The slurry obtained after pretreatment had a total solids content of 11.1% and a WIS content of 7.6%. Almost 100% of the glucose was recovered, based on the content in the raw material, of which about 95% was recovered in the solid fraction (Figure 2). The recovery of xylose, which is the main sugar in hemicellulose, was 67%, the major fraction of which was obtained in the liquid phase (prehydrolysate).

The proportions of glucan and lignin were increased by pretreatment, from 38.8 and 16.1% (Table 2) of the DM in the raw material to 67.6 and 23.1% (Table 3; sugars are presented as monomers) of the DM in the SPWS, respectively, indicating a high degree of hemicellulose hydrolysis. This was confirmed by the xylose concentration of 24.9 g/L in the prehydrolysate, corresponding to 65% of the theoretical yield available in the raw material (Figure 2). Other sugars were also present, mostly in monomer form. Some inhibitors, such as acetic acid, HMF and furfural were also detected after pretreatment (Table 3). The concentrations of acetic acid, HMF and furfural corresponded to yields of 1.2, 0.3 and 1.0 g/100 g dry straw, respectively, which is somewhat higher than the values reported by Linde *et al*. for wheat straw pretreated under the same conditions [14]. The concentration of furfural was higher than that of HMF, because the hemicellulose consisted mostly of pentoses, which is typical of herbaceous crops [34], and the cellulose fraction was barely hydrolysed in steam pretreatment.

**Table 3:** Composition of WIS and liquid (prehydrolysate) fractions in steam-pretreated wheat straw slurry.

Steam-pretreated wheat straw				
**WIS**		**Prehydrolysate**		

**Components**	**Percentage of DM**	**Components**	**Oligosaccharides**	**Monosaccharides**

Glucan	67.6 ± 0.5	**Sugars, g/L**		
Mannan	BDL	Glucose	0.9 ± 0.1	2.3
Xylan	0.7 ± 0.0	Mannose	4.1 ± 0.1	1.1
Galactan	BDL	Xylose	2.9 ± 0.7	22.0
Arabinan	0.4 ± 0.0	Galactose	BDL	1.4
ASL^a^	5.1 ± 0.2	Arabinose	BDL	2.9
AIL^b^	23.1 ± 0.2	**Inhibitors, g/L**		
Total ash	1.0 ± 0.1	Acetic acid	1.7	
AIA^c^	0.4 ± 0.2	HMF	0.4	
		Furfural	1.5	
		Total ash, % of prehydrated material	0.3 ± 0.2	

All the sugars in the prehydrolysate are reported as monomer sugars. Where standard deviations are presented, it was calculated from duplicate measurements on the same sample.

^a^Acid-soluble lignin.

^b^Acid-insoluble lignin.

^c^Acid-insoluble ash.

BDL = below detection limit; DM = dry matter; WIS = water-insoluble solids.

### Enzymatic hydrolysis of wheat meal

It has been shown previously that the amyloglucosidase dosage can be reduced by 5-10% when saccharification is carried out before fermentation [25]. However, a high glucose concentration at the beginning of SSF with lignocellulosics should be avoided to prevent end-product inhibition of the enzymes and osmotic stress to the yeast cells. β-glucosidase activity is reduced by 80% in the presence of only 10 g/L glucose when p-nitrophenyl-β-D-glicopyronoside is used as substrate, and less significantly with cellobiose [35]. In that study also, a high degree of inhibition of cellulase activity was observed at a glucose concentration range of 0 to 100 g/L. Osmotic stress affects the yeast cell when the glucose in the solution is > 150 g/L [25,36]. Therefore, instead of completely saccharifying the wheat meal, we chose to perform partial saccharification (presaccharification). Optimum presaccharification in a starch-based material is about 50-70 dextrose equivalents (DE), which is an indication of the total amount of reducing sugars, expressed as D-glucose, present in the solution [25,37].

**Figure 3:**
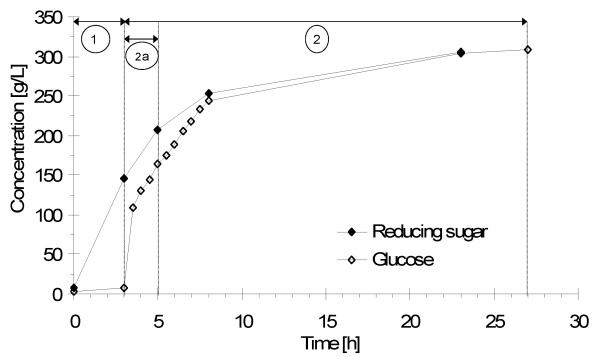
**Starch hydrolysis profiles**. **(1) **Liquefaction, **(2a) **presaccharification and **(2) **saccharification.

In the present study, we achieved 68 DE during starch hydrolysis after a total reaction time of 5 h (liquefaction and subsequent saccharification). The glucose monomer fraction after presaccharification was 53% of the amount of glucose measured at the end of the reaction (Figure 3). The glucose concentration in the PWM after presaccharification was 164 g/L. The PWM was diluted before SSF (Table 1).

### Effect of PWM on the ethanol concentration

Significant differences, in terms of the initial rate of ethanol formation, were observed between SSF on pure SPWS and SSF on mixtures containing different proportions of PWM. During the first 2 hours of SSF, the ethanol productivity was 1.6 g/L/hour in the case of pure SPWS, whereas it was ≥4.7 g/L/hour for PWM alone or PWM mixed with SPWS. This could be due to the high water-soluble sugar content of PWM (Table 1) present at the beginning of fermentation, mainly as glucose, which was consumed rapidly (data not shown), resulting in an increased rate of ethanol formation. In pure SPWS, the major part of the glucose is in polymeric form bound in the solid phase, and this had to be hydrolysed before fermentation. However, glucose was measured in the solution during the initial 8 hours, which means that hydrolysis is not the rate-limiting step in this reaction. However, furfural and HMF may cause a lag-phase in ethanol fermentation [38], because ethanol production is inhibited by the degradation of these compounds to furfuryl alcohol and HMF alcohol, respectively. The most rapid ethanol formation (6.7 g/L/hour) was obtained with pure PWM.

**Figure 4:**
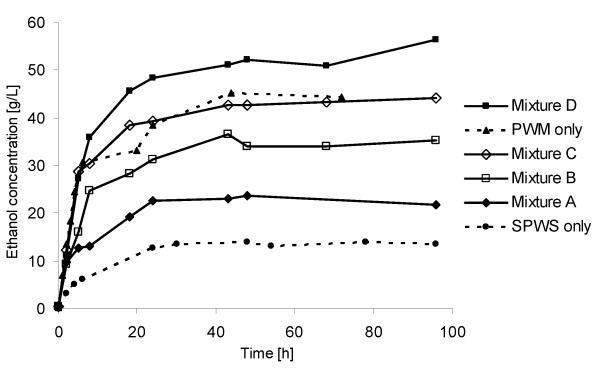
**Variation in ethanol concentration with time during simultaneous saccharification and fermentation (SSF) of steam-pretreated wheat straw (SPWS), presaccharified wheat meal (PWM) and mixtures of the two (A to D)**. PWM to SPWS ratios in the mixtures A to D were 0.8:4.2; 1.5:3.5; 2.0:3.0 and 2.5:2.5, respectively.

Ethanol concentrations increased with increased glucose content in the SSF experiments (Figure 4). The highest concentration obtained was 56.5 g/L (at 96 h) when SSF was carried out on mixture D. Increasing the ethanol concentration is beneficial to the energy demand of distillation [24].

### Effect of PWM on the yield

The ethanol yield is usually reported as g EtOH/g DM of the raw material. However, this means of expressing the yield was not appropriate for this study because mixtures of materials were used. Therefore, the yields are expressed as a percentage of the theoretical maximum, considering only the glucose available in the substrates, as galactose and the pentoses are not usually fermented to ethanol by *S. cerevisiae *[30]. These sugars were not consumed in any of the SSF experiments, which validates this assumption (data not shown).

**Figure 5:**
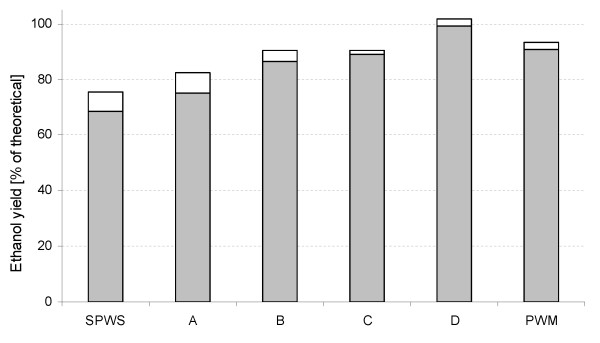
**Ethanol yield (shaded grey areas) based on glucose only in simultaneous saccharification and fermentation (SSF) of pure substrates and their mixtures (A to D)**. Potential ethanol yield after correction for the lactic acid produced from hexoses (white areas).

Figure 5 shows the ethanol yields for the SSF of various mixtures of substrate. The yield from pure SPWS was 68%, which is in agreement with the results obtained by Linde *et al. *[14]. The yield observed for pure PWM was about 91%, which is also typical for SSF of starch-based materials [25]. In mixtures of the two substrates, the yield increased as the proportion of PWM was increased. The highest ethanol yield, 99%, was obtained for SSF of mixture D. This yield is rather high and could be due to some errors in the raw materials analysis. However, this would affect all trials in the same way, thus the most important result is the difference in yield between the experimental points, which shows a clear trend of increasing yield as the ratio of the PWM increased, and most importantly, resulting in higher yield than that from SSF of pure PWM. This is very favourable in terms of process economy and can be explained by the presence of inhibitors at low concentrations (see below).

Small amounts of lactic acid were produced after 40 h of fermentation in all cases. Lactic acid can be formed from both hexoses and pentoses as a microbial metabolic product. The lactic acid yield in this process was 1 g/g consumed sugar regardless of the type of monomeric sugar. The amount of additional ethanol that could have been produced from hexoses if there had not been any lactic acid formation can be estimated as follows:

0.46 g/g is 90% of the maximum theoretical ethanol yield for hexose sugars. After applying this correction for lactic acid, the yield slightly exceeds the theoretical maximum for mixture D (Figure 5).

**Figure 6:**
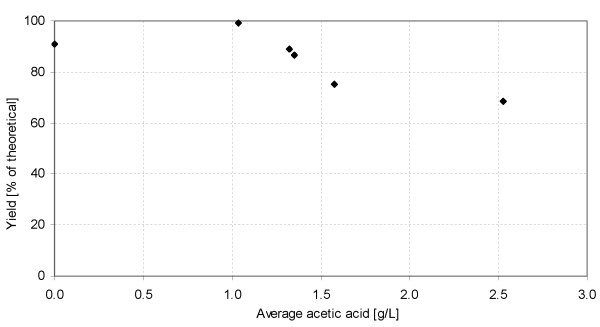
**Ethanol yield as a function of acetic acid concentration during simultaneous saccharification and fermentation (SSF) (average of acetic acid concentrations at 0 and 24 h)**.

Several fermentation inhibitors were present in the slurry during SSF when SPWS was also added to the mixtures. The most significant inhibitors, because of their concentrations, were acetic acid and furfural. Acetic acid is one of the most important weak acids of the fermentation inhibitors, as its pK_a _is close to the pH of fermentation, and therefore a significant amount is in the undissociated form. The undissociated form can diffuse through the plasma membrane, and may dissociate inside the cell, depending on the pH. To avoid the drop in intracellular pH, the cell must pump out protons by the action of the plasma membrane ATPase [39,40]. This means that more ATP has to be generated to maintain the intracellular pH, which is achieved in anaerobic conditions by producing more ethanol, resulting in increased ethanol yield [19,38,41]. Increased ethanol yield has also been noted in the presence of furfural at low concentrations [42]. However, at higher concentrations of acetic acid (> 10 g/L) [18], the ethanol yield is decreased. Similarly, furfural at concentrations of > 3 g/L reduces the ethanol productivity to a great extent [43]. The ethanol yield as a function of the acetic acid concentration in SSF is illustrated in Figure 6. The values given are the average of the concentrations at 0 and 24 h, because acetic acid was released from the hemicellulose fraction at the beginning of SSF. The maximum yield was 99% (obtained at an acetic acid concentration of 1.0 g/L), which is even higher than the yield from PWM fermentation when no acetic acid was present. These results are in good accordance with a previous study reported by Larsson *et al*.; however, those authors expressed the ethanol yield as a function of the total acid concentration [18]. That study also showed that the presence of acetic acid (5 g/L), formic acid (10 g/L), levulinic acid (23 g/L), furfural (1.2 g/L) and HMF (1.3 g/L) only slightly decreased the ethanol yield compared with the reference fermentation. The combination of acetic acid and furfural has been shown to have a negative effect on growth [44]; however, at the low concentrations produced in the present study, they might have a positive effect on ethanol production yield.

The specific raw material demands and the straw to meal ratios for the process scenarios are listed in Table 4. In the pure wheat straw-based process, 5.4 kg dry raw material is needed to produce 1 L of ethanol, whereas the corresponding amount of wheat meal is only 2.4 kg. This is simply because of the difference in the proportions of carbohydrates in the raw materials. The raw material:meal ratios are in the range of 0.5-2.6 in the mixtures.

**Table 4:** Specific raw material demand

Substrate	Raw material required (kg total DM per L EtOH)	Straw:meal
Wheat straw only	5.4	-
Mixture A	4.0	2.6
Mixture B	3.1	1.1
Mixture C	2.9	0.7
Mixture D	2.5	0.5
Wheat meal only	2.4	0

DM = dry matter.

The residue:crop ratio for wheat is typically about 1.3:1.0 (w/w) [45]. Approximately 30-40% of the straw is left on the field for soil protection, leaving the same amount of residue for biomass utilization when the crop is harvested (that is, the straw:wheat ratio is 1.0:1.0). In the case of mixture D, which gave the highest yield, the proportion of wheat meal was double that of the wheat straw. However, the yield was still rather high (87% of theoretical yield) when straw and meal were used in the proportions at harvest, as in mixture B. By comparison, the total yield obtained if the fermentations are carried out separately is 78%.

## Conclusions

In this study, we investigated the effects on ethanol yield of mixing different proportions of PWM and SPWS before SSF. The highest yield was obtained when equal amounts of PWM and SPWS (based on WIS) were used. Thus, a mixed substrate is favourable in terms of final ethanol yield, probably due to the stress on *S. cerevisiae *caused by weak acids present in SPWS. At the same time, it is also easier to reach a high ethanol concentration using such as mixture than when using wheat straw only as a raw material.

Increasing the proportion of WIS of the lignocellulosic material should be studied further in an attempt to improve the ethanol production from mainly lignocellulosics. Bearing in mind the significant proportion of hemicelluloses in wheat straw, a pentose-fermenting yeast should also be considered as a potential alternative. Assuming 70% ethanol yield from pentoses, the final ethanol concentration in the fermentation broth could be further improved by 3-5 g/L ethanol. To decrease the cost of chemicals, decreasing the amount of added nutrients is an option to consider and further investigate when wheat hydrolysate is used as a supplement to SSF with lignocellulosic substrate.

## Competing interests

The authors declare that they have no competing interests.

## Authors' contributions

BE, MG and GZ designed and coordinated the study. ZB and BS carried out the experiments. BE, ZB and BS analysed the results. BE and ZB wrote the paper, and KR, MG and GZ reviewed the paper. All authors read and approved the final manuscript.
